# Paraneoplastic cerebellar degeneration syndrome associated with breast cancer: a case report

**DOI:** 10.1002/alz70860_107717

**Published:** 2025-12-23

**Authors:** Glauco Kody Nagata, Eduarda Rosa Fernandes, Tatiane Morgana da Silva, Carolina Heck Haubrich, Lucas Porcello Schilling, Cristiano Aguzzoli

**Affiliations:** ^1^ Neurology Department, São Lucas Hospital of PUCRS, Porto Alegre, Rio Grande do Sul, Brazil; ^2^ Brain Institute of Rio Grande do Sul (InsCer), Porto Alegre, Rio Grande do Sul, Brazil

## Abstract

**Background:**

We present a 66‐year‐old female with persistent subacute vertigo lasting 3 weeks, along with a 15 kg weight loss. Her medical history included rheumatoid arthritis, type 2 diabetes, gastroesophageal reflux disease, hypertension, anxiety, and depression. Initially, she was discharged from the emergency department and referred to a neurology outpatient clinic. Six months later, during a follow‐up examination, she exhibited opsoclonus, tongue fasciculations, dysmetria with bilateral intentional tremor, tactile hypoesthesia in the limbs, and significant ataxia.

**Method:**

Hospitalized for further evaluation, she underwent extensive testing. Magnetic Resonance Imaging (MRI) revealed cerebellar atrophy. Cerebrospinal fluid (CSF) analysis showed oligoclonal bands and an IgG index of 6.2, while other results were normal. Pulse therapy with methylprednisolone failed to improve her symptoms. A chest CT scan revealed a left axillary lymph node (1.4 cm x 1.4 cm), and breast MRI was classified as BI‐RADS 5. A wholebody PET‐CT scan showed hypermetabolic activity in the lymph node, suggesting malignancy.

**Result:**

The patient underwent a left lymph node biopsy, and immunohistochemical analysis revealed: positive CK 7 (SP 52), negative CK 20 (Ks 20.8), positive GATA‐3 (L50‐823), weakly positive estrogen receptor (ER) in 2% of cells, negative progesterone receptor (PR), indeterminate C‐erbB‐2, and a Ki‐67 proliferative index of 50%. These findings confirmed metastasis from primary breast carcinoma.

**Conclusion:**

The diagnosis of paraneoplastic syndrome with cerebellar degeneration secondary to breast cancer was established. Paraneoplastic neurological syndromes, triggered by autoimmune responses to tumors or metastases, may present months or even years before or after cancer diagnosis. Early recognition and treatment are essential not only to manage neurological symptoms but also to identify and treat the underlying malignancy. This case underscores the importance of comprehensive clinical evaluation for differential diagnosis of neurological syndromes.

